# FAST BEE (Keeping FAST First): A Public Stroke Education Design to Drive EMS Activation

**DOI:** 10.1002/cns.71003

**Published:** 2026-06-29

**Authors:** Renyu Liu, Jing Zhao, Siju V. Abraham, Gary A. Ford

**Affiliations:** ^1^ Department of Anesthesiology and Critical Care, and Neurology Perelman School of Medicine at the University of Pennsylvania Philadelphia Pennsylvania USA; ^2^ Department of Anesthesiology and Perioperative Medicine Robert Wood Johnson University Hospital, Rutgers University New Brunswick New Jersey USA; ^3^ Department of Neurology Minhang Hospital Affiliated to Fudan University Shanghai China; ^4^ Department of Emergency Medicine Jubilee Mission, Medical College & Research Institute Thrissur Kerala India; ^5^ Radcliffe Department of Medicine University of Oxford Oxford UK

## Abstract

Improving stroke recognition and promoting immediate action are critical to enhancing prehospital stroke care. For two decades, the FAST (Face, Arm, Speech, Time) mnemonic has been widely taught, yet it fails to capture many posterior circulation strokes. To address these limitations, BE FAST (adding Balance and Eye symptoms) was introduced but showed reduced recall for more common symptoms represented by FAST, likely due to common symptoms being placed after less common ones, thus weakening retention of the original FAST elements. To improve both detection and action, we propose **FAST BEE**—keeping FAST first and adding BE after FAST with an additional “E” for Emergency Medical Services (EMS) call. This structure reinforces the urgency of activating EMS by repeating the call‐to‐action within the mnemonic. We further introduce a novel **FAST BEE cartoon** as a visual tool to enhance learning, recall across diverse populations, and immediate action (EMS call) in public. Using the bee metaphor provides a memorable, engaging, and globally adaptable educational strategy in English‐speaking populations. FAST BEE offers a fresh, action‐oriented framework that builds on the familiarity of FAST, emphasizes immediate EMS activation, and holds promise to strengthen stroke action awareness worldwide. Further evaluation of effectiveness in various English‐speaking populations is needed.

## Graphical abstract

FAST BEE is a public stroke education design that intentionally keeps the widely recognized FAST framework first (Face drooping, Arm weakness, Speech difficulty, and Time to call EMS) while adding Balance symptoms, Eye (vision) symptoms, and reinforced EMS activation. BEE functions as a memorable educational cue that encourages recognition of stroke symptoms beyond FAST without replacing or altering the established FAST message. By preserving familiarity while broadening symptom recognition, FAST BEE aims to improve stroke action awareness, increase EMS activation, facilitate earlier treatment, and reduce stroke‐related mortality and disability.

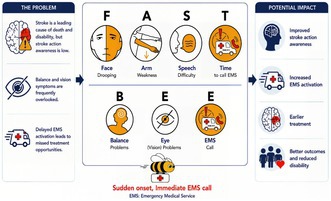

Improving stroke recognition and prompting immediate action remain top priorities for enhancing prehospital stroke care [1, 2]. For two decades, the public has been taught the acronym **FAST**—Face, Arm, Speech, Time—to help identify the most common stroke symptoms and call emergency services without delay. The FAST signs (facial droop, arm weakness, and speech disturbance) may be absent in a substantial proportion of ischemic strokes. In the seminal BE‐FAST study, 14.1% of ischemic stroke patients lacked FAST findings, and 71% of FAST‐missed strokes involved the vertebrobasilar circulation [3]. Furthermore, posterior circulation stroke patients are more likely to be FAST‐negative and consequently less likely to receive timely reperfusion therapy, highlighting the need for public recognition tools that better capture balance and visual symptoms [3, 4].

To capture these missed cases, the **BE FAST** acronym was developed, adding **B** for balance difficulties and **E** for eye symptoms [3]. Although BE FAST aimed to increase detection, recent studies show it does not improve recall compared with FAST [4, 5]. In fact, memory for the original FAST symptoms declined significantly when “BE” was placed before FAST. People often recall the first items in a sequence best, and changes in item order may influence retention. Placing “BE” at the start may unintentionally weaken recall of the original, critical FAST elements. To strengthen both detection and action without displacing the familiar sequence, we propose FAST BEE—keeping FAST first, appending B (Balance) and E (Eye symptoms), and adding a final E for Emergency call, while reframing T as “Time to call EMS.” This deliberate repetition targets the behavior (activate EMS) and will be evaluated in preregistered trials. FAST BEE preserves FAST (Face, Arm, Speech, T = Time to call EMS) and appends BEE—B = Balance difficulties; E = Eye symptoms (sudden vision loss or blurring); E = Emergency call (call EMS)—thereby repeating the call‐to‐action. By repeating the call‐to‐action and pairing it with a simple visual bee metaphor, FAST BEE aims to strengthen both symptom recognition and timely EMS activation.

We also introduce a FAST BEE cartoon (Figure 1) as a memorable visual aid across ages, with the aim of strengthening learning, long‐term recall, and immediate action. To reinforce the importance of calling for help, the repeated structure is used—once with the **T** (“Time to call the Emergency Medical Services (EMS)”) and again with the last **E** (“EMS call”). For country‐specific applications, the local EMS phone number can be added or substituted for T and E, as illustrated. This cartoon was developed for countries where 911 is the emergency number, aligning with the Stroke 911 program for non‐English speaking people [6]. To support comparison and implementation, we produced a brief video illustrating FAST BEE (Video S1) [7]. The video can be dubbed, subtitled, and combined with other formats—posters, radio spots, and short social videos—to match local contexts and broaden dissemination across countries. Planned media variants include radio jingles and short rap segments tailored for schools and community channels. Notably, short cartoon animations broadcast on television were successfully used in China as part of a stroke action–awareness campaign and were associated with a significant reduction in prehospital delay [8]. Similar approaches using innovative art forms were successfully used in stroke education [9, 10].

**FIGURE 1:**
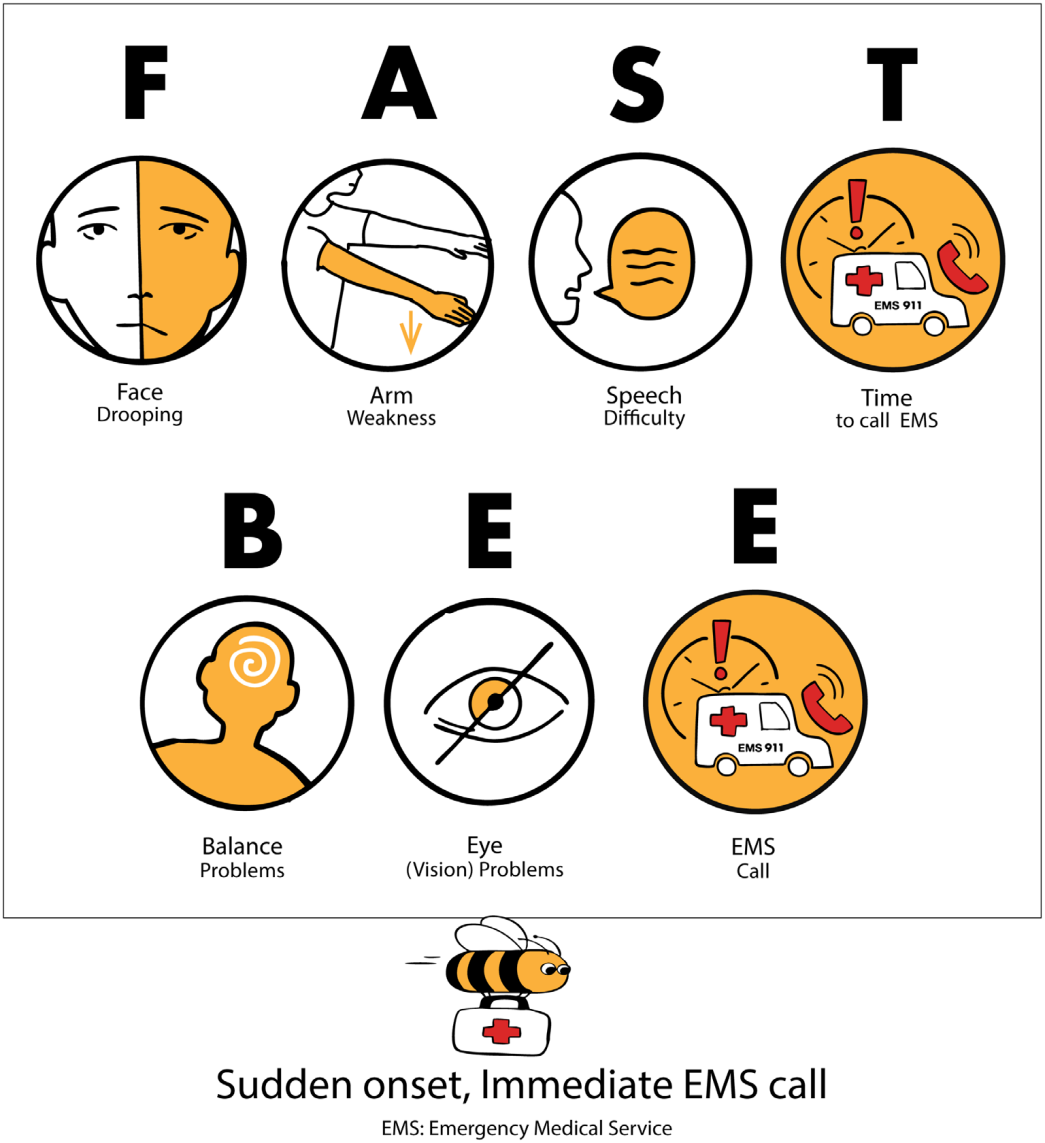
FAST BEE mnemonic. FAST: Face droop; Arm weakness; Speech difficulty; T = Time to call EMS. BEE: B = Balance difficulties; E (first) = Eye problems (vision loss or blurring); E (second) = Emergency Medical Services (EMS) call. Here, 911 is shown for countries where 911 is used, such as the United States and Canada. It can be replaced with other EMS numbers like 000, 999, or 112. The original design of the cartoon is available from the corresponding author upon request. Cartoon credit: Lilian Liu.

Human memory is wired to remember stories and vivid imagery more effectively than plain lists. Mnemonics that draw on nature or relatable metaphors are easier to recall and share. Research shows that *situated mental imagery*—imagining oneself interacting with meaningful elements—significantly boosts memory retention [11]. FAST BEE uses the bee metaphor to create a vivid, relatable context for speed, balance, and coordinated action that might be useful in early childhood education. By engaging children early, we can lay the foundation for lifelong stroke awareness and potentially reduce the global stroke burden. We plan to perform school‐based studies to evaluate whether the bee metaphor aids recall and engagement among children. School‐based studies, followed by learner‐to‐family activities, may effectively extend knowledge to households [12, 13].

Like bees that labor to keep their honey‐sweet hives thriving and move quickly when danger nears, human communities should act decisively at the first sign of stroke—call the emergency number immediately to protect our happy communities; potential public slogans (localize “EMS/emergency number,” e.g., 911/112/999/000) include: “Sudden onset? FAST BEE—buzz into action. Call EMS.”; “Be like a bee: act fast. Call EMS at the first stroke sign.”; and—adult‐facing if “sting” is acceptable—“Sudden onset? FAST BEE—sting into action. Call EMS.”

For public education, FAST BEE offers an incremental strategy: it reinforces the globally established “FAST” message while introducing additional elements for those able to retain more information. The “BEE” component can also function independently as a complementary tool to FAST to emphasize specific stroke symptoms and the importance of calling EMS. Therefore, in designing the cartoons in Figure 1A, we adopted a two‐row layout strategy. This approach strengthens stroke recognition and timely EMS activation by deliberately repeating “T” and “E” to cue the specific behavior: “Time to call EMS.” The apparent redundancy is intentional and aligns with our shift from “stroke awareness” to “stroke action awareness.” In public messaging, T in FAST is often interpreted variably (e.g., “time is brain” or simply “time”), which signals urgency but not the required action. We therefore reframe T as “Time to call”/“Telephone” and make the final E as “Emergency call” explicit to prevent common errors (e.g., calling family, a primary physician, or self‐transport). This guided repetition reduces action ambiguity and reinforces the target behavior (e.g., “Time to call EMS—call EMS now!”). The authors acknowledge a potential trade‐off from adding extra letter E and will evaluate the benefit of the dual action cue, testing for any net effect on recall versus action.

Acronyms like “FAST” work well in English but are less effective where English is not widely spoken. FAST BEE may pose even greater challenges across languages because it adds letters to remember. Accordingly, FAST‐BEE is intended primarily for English‐speaking populations. We will collaborate with society leaders, local experts, and stakeholders to co‐develop culturally and linguistically appropriate adaptations and evaluate their effectiveness. We have piloted local adaptations of FAST in China, Mexico, and Nigeria, providing cultural experience and a solid foundation for adapting FAST BEE across diverse regional environments [8, 14, 15]. In non‐English settings, FAST BEE can function as a visual adjunct rather than replace established tools; for example, in Spanish‐speaking populations, RÁPIDO can remain the verbal mnemonic, while the bee icon—signaling swift, decisive action—and an explicit emergency‐call cue reinforce the action step [16, 17].

As the sequence of letters changes, and the number of letters to remember increases, a vivid cartoon mascot may help memory retention. Rigorous evaluation is under preparation or ongoing for comparative trials (FAST vs. BE FAST vs. FAST BEE) measuring immediate recall, 1–3‐month retention, intent to call EMS, and performance in simulations, including cultural cohorts in various countries. The innovative FAST4D scoring system (diplopia, visual‐field deficit, dizziness/vertigo, dysmetria/ataxia) will inform our trial design and analysis [18].

FAST BEE introduces additional information and a repeated letter (“E”), which may either reinforce action‐oriented behavior or increase cognitive complexity. Whether the benefits of added symptom recognition and action reinforcement outweigh the potential reduction in mnemonic simplicity remains unknown and will require prospective comparative testing. We further acknowledge that FAST BEE may ultimately prove inferior to FAST or BE FAST in recall performance; therefore, comparative testing rather than theoretical reasoning should determine its future role in public stroke education.

FAST BEE offers a fresh, memorable, and action‐focused way to improve stroke recognition. By repeating the call‐to‐action and embedding it in a powerful visual metaphor, FAST BEE could strengthen both awareness and emergency action. However, FAST BEE is not intended as a replacement for FAST at this stage. Rather, it is presented as a hypothesis‐generating educational design that seeks to preserve the familiarity of FAST while exploring whether posterior circulation symptom recognition and EMS activation can be further improved. Determining whether this balance can be achieved is precisely the question we plan to study for a clear answer. Testing this approach across diverse English‐speaking populations is needed to determine its impact on the public response to stroke symptoms.

## Funding

This work was supported by the University of Pennsylvania (Grant 0000000115).

## Conflicts of Interest

The authors declare no conflicts of interest.

## Supporting information


**Video S1:** FAST BEE: Video demonstration of stroke recognition and emergency response. This video illustrates the FAST BEE mnemonic for public stroke recognition. The animation demonstrates recognition of both anterior‐ and posterior‐circulation stroke symptoms, including facial droop, arm weakness, speech disturbance, balance impairment, and visual abnormalities, while emphasizing the importance of immediate activation of emergency medical services. The video was designed as a public education tool to improve stroke action awareness and facilitate timely hospital arrival and reperfusion treatment. The version presented in this video is intended for regions where **911** serves as the emergency telephone number. The emergency contact number can be readily adapted to local emergency medical service (EMS) systems in other countries and regions without altering the core educational content of the FAST BEE mnemonic.

## Data Availability

This article is an editorial commentary and does not report original research data. The educational materials referenced, including graphics and video resources related to the FAST BEE initiative, are available from the corresponding author (renyu.liu@pennmedicine.upenn.edu) upon reasonable request.
